# Molecular structure of a 5,10‐methylenetetrahydrofolate dehydrogenase from the silkworm *Bombyx mori*

**DOI:** 10.1002/2211-5463.12595

**Published:** 2019-02-26

**Authors:** Mohammad R. Haque, Akifumi Higashiura, Atsushi Nakagawa, Aiko Hirowatari, Shigeki Furuya, Kohji Yamamoto

**Affiliations:** ^1^ Department of Bioscience and Biotechnology Kyushu University Graduate School Fukuoka Japan; ^2^ Institute for Protein Research Osaka University Suita Japan; ^3^ Present address: Department of Virology Graduate School of Biomedical and Health Sciences Hiroshima University Hiroshima Japan

**Keywords:** 5,10‐methylenetetrahydrofolate dehydrogenase, *Bombyx mori*, crystal structure, NADP, serine

## Abstract

The enzyme 5,10‐methylenetetrahydrofolate dehydrogenase (MTHFD) is essential for the production of certain amino acids (glycine, serine, and methionine) and nucleic acids (thymidylate and purine). Here, we identified a cDNA encoding this enzyme from the silkworm *Bombyx mori*. The recombinant *B. mori* MTHFD (bmMTHFD) expressed in *Escherichia coli* recognized 5,10‐methylenetetrahydrofolate and 5,10‐methenyltetrahydrofolate as substrate in the presence of NADP ^+^ as well as NAD ^+^. The bmMTHFD structure was determined at a resolution of 1.75 Å by X‐ray crystallography. Site‐directed mutagenesis indicated that the amino acid residue Tyr49 contributed to its catalytic activity. Our findings provide insight into the mechanism underlying the activity of MTHFD from *B. mori* and potentially other insects and may therefore facilitate the development of inhibitors specific to MTHFD as insecticides.

**Abbreviations**
bmMTHFD: *Bombyx mori* 5,10‐methylenetetrahydrofolate dehydrogenase
C: cyclohydrolase
D: dehydrogenase
MTHF: 5,10‐methylenetetrahydrofolate
MTHFD: 5,10‐methylenetetrahydrofolate dehydrogenase
S: synthetase
WT: wild‐type

One‐carbon metabolism is involved in the synthesis of amino acids like alanine, glycine, and serine, as well as purine and pyrimidine bases 1, 2. 5,10‐methylenetetrahydrofolate (MTHF) dehydrogenase (MTHFD; EC1.5.1.5) is an enzyme involved in one‐carbon metabolism in eukaryotes 2. To date, cytosolic and mitochondrial versions of this protein have been reported. For example, MTHFD1 is a cytoplasmic protein 3, whereas MTHFD1L, MTHFD2, and MTHFD2L are mitochondrial proteins 4, 5, 6. Human MTHFD1 is a trifunctional enzyme with dehydrogenase (D), cyclohydrolase (C), and synthetase (S) activities that catalyze the oxidation of MTHF to 5,10‐methenyl‐THF, which is then hydrolyzed to 10‐formyl‐THF, and finally converted to THF and formate 3. The 3D structure of the D/C domain of MTHFD1, referred to as DC301, has been reported 3. MTHFD2 and MTHFD2L are bifunctional enzymes 7, 8, whereas MTHFD1L is a monofunctional enzyme 9. MTHFD often requires NADP^+^ or NAD^+^ as the cofactor for their activity. MTHFD1 requires NADP^+^ 3, MTHFD2 and MTHFD2L use either NADP^+^ or NAD^+^ 7, 8, whereas MTHFD1L is monofunctional with only S activity and does not use either cofactors 9. Likewise, the prokaryotic MTHFD of *Escherichia coli* is a bifunctional enzyme that uses NADP^+^ 10, and the monofunctional enzyme of *Peptostreptococcus productus* requires NADP^+^ as the cofactor 11. Although one‐carbon metabolism has been studied in vertebrates, there are no reports from invertebrates, including silkworm and other insects.

To characterize one‐carbon metabolism in insects, we isolated mRNA encoding an MTHFD of the silkworm *Bombyx mori* MTHF dehydrogenase (bmMTHFD), which is an important lepidopteran insect model. The structure–function relationships of insect MTHFDs have not been studied in detail. Since many agricultural pests are lepidopteran insects, it is useful to investigate the amino acid residues present in the active site of bmMTHFD. Further, because MTHFD is involved in the synthesis of important biomolecules such as amino acids and purine and pyrimidine bases, the inhibitors could be effective insecticides against agricultural pests. Here, we determined the three‐dimensional structure of bmMTHFD to identify the amino acid residues important for bmMTHFD activity and conducted mutation analysis of bmMTHFD to determine the role of the amino acids lining the substrate‐binding site. Examination of bmMTHFD catalytic activity indicated that it participates in the D and C activities. The active site in bmMTHFD was then determined to better understand the structural basis for this conversion. As described, mammalian MTHFDs are key enzymes involved in the synthesis of amino acids and purine and pyrimidine bases, which are crucial biomaterials for survival. Analysis of inhibition of insect MTHFDs would aid in the design of pesticides and insecticides. The crystal structure of bmMTHFD and the identification of the amino acid residues involved in catalytic function in the current study may provide insights into the mechanism underlying MTHFD activity and could facilitate the development of inhibitors specific to MTHFD as insecticides. To the best of our knowledge, this study is the first to report on MTHFD in insects.

## Materials and methods

### Insects

*Bombyx mori* larvae (p50T strain) were reared at the Kyushu University Graduate School (Fukuoka, Japan) and fed mulberry leaves. Day‐3 fifth‐instar larvae were dissected on ice, and fat body was stored at −80 °C until use.

### RNA extraction, cloning, and sequencing of cDNA encoding bmMTHFD

Total RNA was isolated from the fat body using RNeasy Plus Mini Kit (Qiagen, Hilden, Germany) and was analyzed by reverse transcription–PCR. First‐strand cDNA was obtained using SuperScript II reverse transcriptase (Invitrogen, Carlsbad, CA, USA) and an oligo‐dT primer. The resulting cDNA was used as a PCR template with the following oligonucleotide primers: 5′‐CAACAGCCATATGGCGCGTATCCTCGATGG‐3′ (sense) and 5′‐CCGGATCCTTAATTGGATTTGTTTGCTTGA‐3′ (antisense). The primer designs were based on a partial sequence obtained from the SilkBase database (http://silkbase.ab.a.u-tokyo.ac.jp/cgi-bin/index.cgi). The underlined and double‐underlined regions indicate NdeI and BamHI restriction enzyme sites, respectively, which were used for insertion of the PCR product into an expression vector. The PCR program was as follows: 94 °C for 2 min, 35 cycles of 94 °C for 1 min, 59 °C for 1 min, and 72 °C for 2 min, and 72 °C for 10 min. The bmMTHFD cDNA (*bmmthfd*) was ligated into the pGEM‐T Easy Vector (Promega, Madison, WI, USA) and transformed into *E. coli* DH5α.

To obtain the complete sequence of *bmmthfd* and to deduce its amino acid sequence, the GENETYX‐MAC software (ver. 14.0.12; GENETYX Corporation, Tokyo, Japan) was used. Homology alignment was performed using clustalw (ver. 1.83; DNA Data Bank of Japan, Shizuoka, Japan), with 10 and 0.2 as the gap creation penalty and gap extension, respectively. A phylogenetic tree was generated using neighbor‐joining plot software (http://doua.prabi.fr/software/njplot).

### Overexpression and purification of recombinant protein

The *bmmthfd* clone was digested with NdeI and BamHI, subcloned into the expression vector pET‐15b (Merck Millipore, Darmstadt, Germany), and transformed into competent *E. coli* Rosetta (DE3) pLysS cells (Merck Millipore). The cells were then grown at 37 °C in Luria–Bertani media containing 100 μg·mL^−1^ ampicillin. After the cell density reached an OD_600_ of 0.7, isopropyl‐1‐thio‐β‐d‐galactoside was added to a final concentration of 1 mm to induce recombinant protein production. The culture was further incubated for 3 h, and the cells were harvested by centrifugation at 10 000 ***g*** for 15 min. Bacteria were resuspended in phosphate‐buffered saline (137 mm NaCl, 2.7 mm KCl, 10 mm Na_2_HPO_4_, and 1.76 mm KH_2_PO_4_, pH 7.4) containing 4 mg·mL^−1^ lysozyme and were subsequently disrupted by sonication. Sonication was performed three times for 1 min each on ice using a 3‐mm tapered microtip probe. The supernatant containing the recombinant protein was clarified by centrifugation at 10 000 ***g*** for 15 min and subjected to Ni^2+^‐affinity chromatography equilibrated with the same buffer. After washing with the same buffer, the samples were eluted with a linear gradient of 0–0.5 m imidazole. The enzyme‐containing fractions, assayed as described below, were pooled, concentrated using a centrifugal filter (10 000 molecular weight cutoff; Millipore Corp., Billerica, MA, USA), and applied to a Superdex 200 column (GE Healthcare Bio‐Sciences, Buckinghamshire, UK) equilibrated with 20 mm Tris/HCl buffer (pH 8.0), with the addition of 0.2 m NaCl. The purity of the pooled material was analyzed by sodium dodecyl sulfate (SDS)/PAGE performed using a 15% polyacrylamide slab gel containing 0.1% SDS, according to the method reported by Laemmli 12. Protein bands were visualized by Coomassie Brilliant Blue R‐250 (Sigma‐Aldrich, St. Louis, MO, USA) staining.

### Protein crystallization and X‐ray diffraction data analysis

Recombinant bmMTHFD was purified as described in ‘Overexpression and purification of recombinant protein’ and then concentrated using a centrifugal filter (Millipore) to 10 mg·mL^−1^ in 20 mm Tris/HCl buffer, pH 8.0, containing 0.2 m NaCl. Crystallization was performed using the sitting‐drop vapor diffusion method at 20 °C with Crystal Screen Kits (Hampton Research, Aliso Viejo, CA, USA). Each drop was formed by mixing an equal (1 μL) or twofold greater volume (1 : 2 μL) of protein and reservoir solutions, respectively. Crystals suitable for X‐ray analysis were grown for 4 weeks in 0.2 m ammonium sulfate, 0.1 m sodium cacodylate trihydrate, and 30% PEG 8000 (w/v). The crystal was transferred to reservoir solution with 25% (v/v) ethylene glycol using a cryoloop and flash frozen with liquid nitrogen before data collection. X‐ray diffraction data collection was performed using synchrotron radiation on SPring‐8 beamline BL44XU with a wavelength of 0.9 Å, in a nitrogen vapor stream at 100 K 13. The data set was integrated and scaled using the denzo and scalepack programs, as implemented in the hkl2000 program package 14.

### Determination of structure

The initial structure was determined using a molecular replacement method with the program phenix phaser‐mr 15, using the polyalanine model of *E. coli* MTHFD (PDB ID: 1B0A) as a search model. The arp/warp program 16 was used to build missing side‐chain atoms and to add water molecules. After manual adjustment to the electron density map with the program coot 17, refinement was performed using the program phenix.refine 18. Figures were prepared using the PyMOL software (http://pymol.sourceforge.net). The atomic coordinates and structure factor of bmMTHFD have been deposited in the Protein Data Bank (PDB ID: 5ZF1). The 3D structure alignment was performed using the matras server 19.

### Site‐directed mutagenesis

Amino acid‐substituted mutants of bmMTHFD were constructed using the QuikChange Site‐Directed Mutagenesis Kit (Agilent, Santa Clara, CA, USA), according to the previous method 20. The mutagenesis primers were as follows: 5′–TAATTGTTGGCGATGACCCAGCGGCTCACACCTATGTCAGGAACAA–3′ (sense) and 5′–TTGTTCCTGACATAGGTGTGAGCCGCTGGGTCATCGCCAACAATTA–3′ (antisense) for S46A, 5′–GATGACCCAGCGAGCCACACCGCTGTCAGGAACAAAGTTGAAGCCGCA–3′ (sense) and 5′–TGCGGCTTCAACTTTGTTCCTGACAGCGGTGTGGCTCGCTGGGTCATC–3′ (antisense) for Y49A, 5′–GCCACACCTATGTCAGGAACGCTGTTGAAGCCGCAAAATTTGT–3′ (sense) and 5′–ACAAATTTTGCGGCTTCAACAGCGTTCCTGACATAGGTGTGGC–3′ (antisense) for K53A, 5′–GTGTTGATGGAATTCTCGTCGCTTTACCTGTACCTGATACAAT–3′ (sense) and 5′–ATTGTATCAGGTACAGGTAAAGCGACGAGAATTCCATCAACAC–3′ (antisense) for Q97A, and 5′–ATTGCTCCTGAAAAAGATATCGCTGGTTTCCACATAATCAACATC–3′ (sense) and 5′–GATGTTGATTATGTGGAAACCAGCGATATCTTTTTCAGGAGCAAT–3′ (antisense) for D120A. An expression plasmid containing *bmmthfd* cDNA was used as a template, and full‐length mutated cDNAs were verified by DNA sequencing with 3730xl DNA Analyzer (Applied Biosystems, Foster City, CA, USA).

### Measurements of enzyme activity

Dehydrogenase activity of bmMTHFD was spectrophotometrically measured using MTHF (Toronto Research Chemicals, North York, ON, Canada) as substrate and NADP^+^ or NAD^+^ as cofactor. MTHF, NADP^+^, and NAD^+^ were stored at −80 °C until use. The reaction mixture (100 μL) included 20 mm sodium phosphate buffer (pH 6.5), 30 mm 2‐mercaptoethanol, and various concentrations of substrate, NADP^+^ or NAD^+^. The assay was initiated by addition of the enzyme and then incubated at 28 °C for 10 min. Production of methenyltetrahydrofolate was measured at 350 nm by an endpoint assay and a background rate determined in enzyme‐free assays. The substrate concentrations were calculated using the extinction coefficient of 24.9 mm ^−1·^cm^−1^ 21. Kinetic parameters were measured with a nonlinear least squares data fit to the Michaelis–Menten equation using kaleidagraph (Synergy Software; HULINKS Inc., Tokyo, Japan).

Cyclohydrolase activity of bmMTHFD was assayed with 5,10‐methenyl‐THF (Santa Cruz Biotechnology, Inc., Dallas, TX, USA), that was stored at −80 °C until use. Reaction mixture (100 μL) contained 0.1 m potassium maleate (pH 7.4), 0.02 m 2‐mercaptoethanol, and 0.1 mm 5,10‐methenyl‐THF. The reaction was started by addition of the enzyme and then incubated at 30 °C for 10 min. The assay was performed by measuring the decrease in absorption at 355 nm 22, and the values were compared to those from an enzyme‐free assay.

## Results

### Sequence of cDNA encoding bmMTHFD

**Figure 1:**
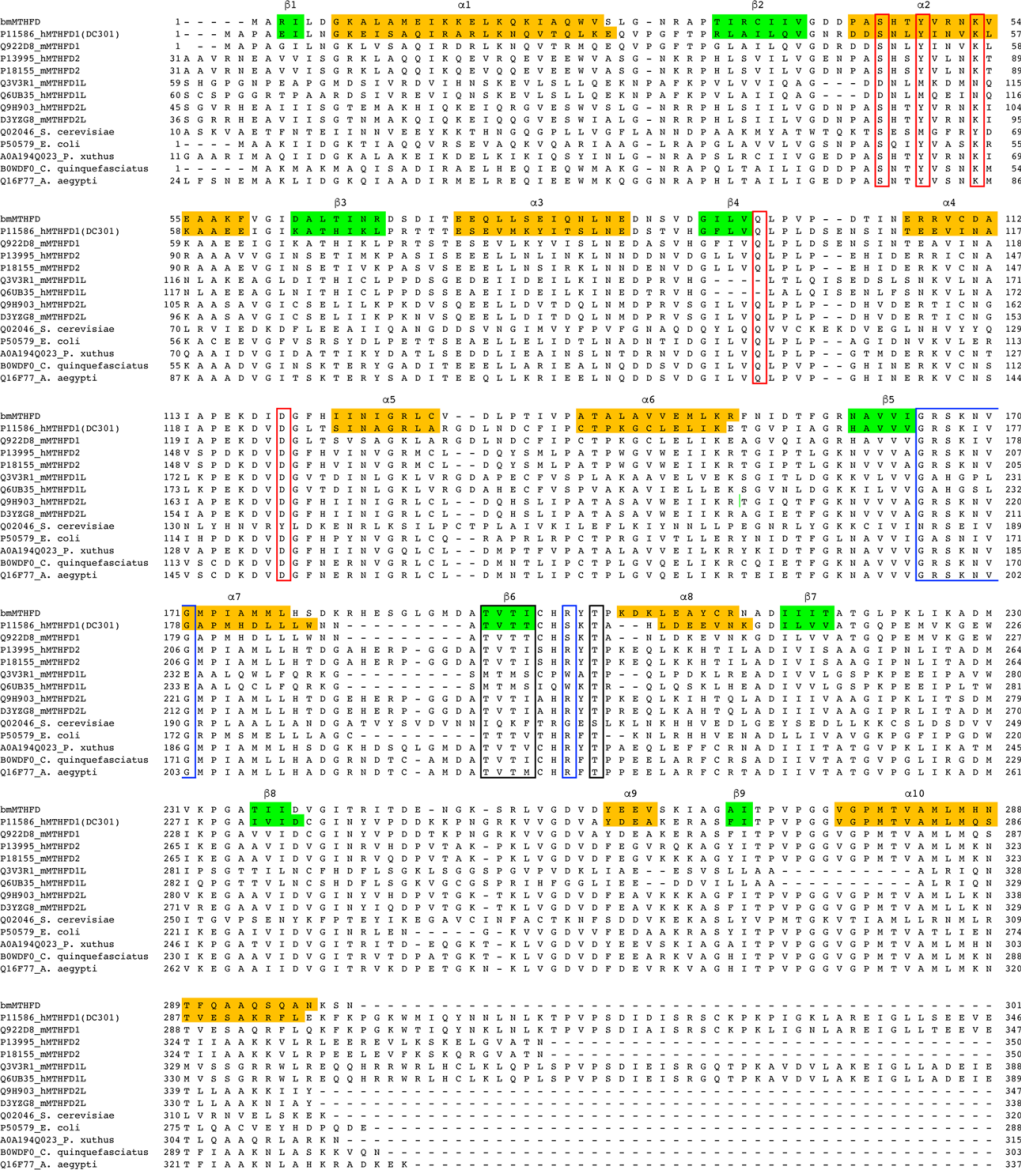
Alignment of amino acid sequences of MTHFDs. Sequences were obtained from the Swiss‐Prot database (http://expasy.org/sprot/). Each entry contains an accession number. DC301 is the D/C domain of hMTHFD1. The NAD(P)^+^‐binding, substrate‐binding, and dimer‐interaction sites are boxed in blue, red, and black, respectively. α‐Helices (shaded in orange) and β‐strands (shaded in green) are labeled by α and β, respectively.

**Figure 2:**
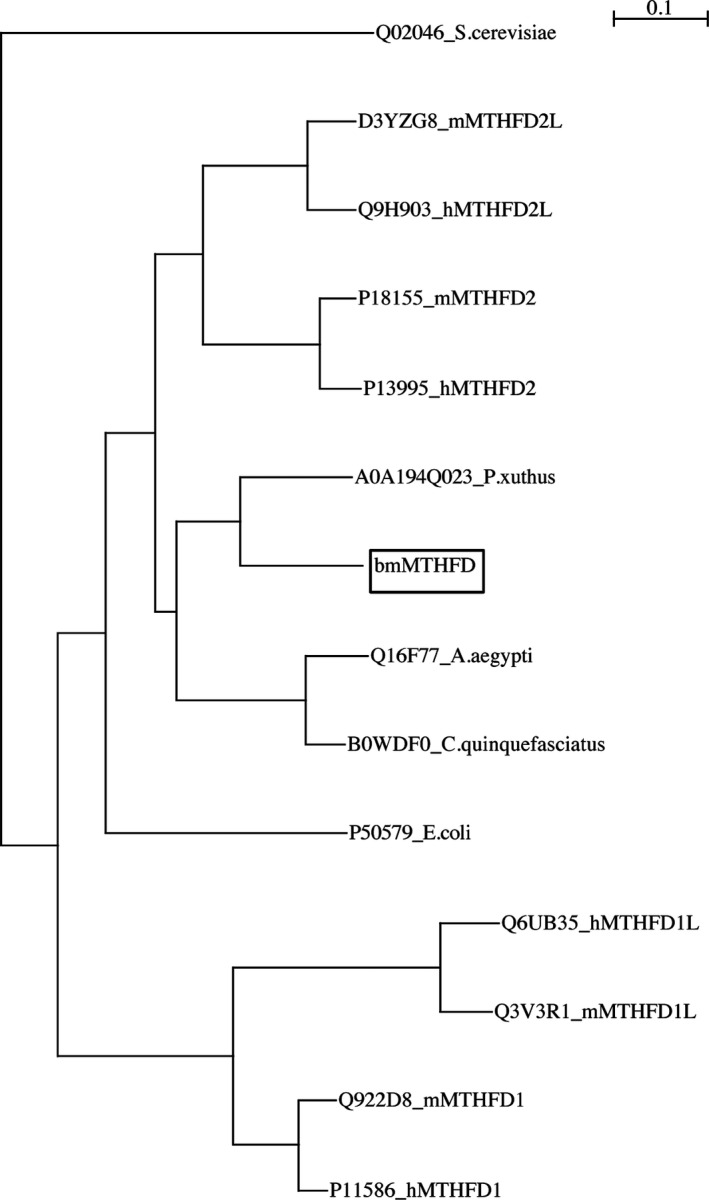
Phylogenetic analysis of MTHFD amino acid sequences. The phylogenetic tree was constructed with a neighbor‐joining plot software using different GST sequences from the Swiss‐Prot database (https://web.expasy.org/docs/swiss-prot_guideline.html). Each entry contains the species name or protein name and the accession number. The numbers attached to the nodes indicate the bootstrap values. bmMTHFD is boxed. The scale bar corresponds to a 0.1 distance.

We isolated *bmmthfd* cDNA from *B. mori,* and the nucleotide sequence has been deposited in DDBJ/EMBL/GenBank databases under accession no. LC366023. The sequence contained an open reading frame of 906 base pairs encoding 301 amino acid residues (Fig. 1). The theoretical molecular mass and isoelectric point of bmMTHFD are 32 733 Da and 6.1, respectively. Screening the predicted 3D model of MTHFD in Protein Data Bank (https://www.rcsb.org) showed highest similarity to the human MTHFD1 with *E*‐value of 1.57E‐92. The amino acid sequence of bmMTHFD showed 41% identity to DC301, 49% to human MTHFD2 (hMTHFD2), 49% to mouse MTHFD2 (mMTHFD2), 23% to *Saccharomyces cerevisiae* MTHFD, 13% to mouse MTHFD1L (mMTHFD1L), 13% to human MTHFD1L (hMTHFD1L), 14% to mouse MTHFD1 (mMTHFD1), 51% to human MTHFD2L (hMTHFD2L), 51% to mouse MTHFD2L (mMTHFD2L), 45% to *E. coli* MTHFD, 72% to *Papilio xuthus* MTHFD, 61% to *Culex quinquefasciatus* MTHFD, and 55% to *Aedes* *aegypti* MTHFD. The DC301 structure has NADP^+^‐ and substrate‐binding sites along with the dimer‐interaction site 3, all of which were conserved among these protein sequences (Fig. 1). Based on the phylogenetic tree generated from the aligned amino acid sequences of the MTHFDs, the bmMTHFD cloned in this study belonged to an independent group including putative MTHFDs of insects (Fig. 2).

### Structural description of bmMTHFD

The crystal structure of bmMTHFD was solved by the molecular replacement method, and the structure was refined against 1.75 Å resolution data. The data collection and refinement statistics are summarized in Table 1. Two molecules were included in the crystallographic asymmetric unit and showed similar structures with root‐mean‐square deviation (rmsd) between the corresponding atoms of 1.05 Å. The residues 44–46 and 246–253 of the A chain and 244–252 of the B chain were not modeled because of poor electron density. Figure S1 shows that bmMTHFD eluted at 205 mL, corresponding to a molecular size of 63 kDa, and appears to be a homodimeric protein based on the elution profile. Dali search for protein structure comparison between the determined structure and structures already deposited in the Protein Data Bank 23 revealed that DC301 (PDB ID: 1A4I) with rmsd of 1.40 Å and hMTHFD2 (PDB ID: 5TC4) with rmsd of 1.10 Å were most similar. The sequence alignment of bmMTHFD to that of DC301 showed that the α‐helices and β‐strands of bmMTHFD were conserved across the structures (Fig. 1). bmMTHFD is composed of two domains containing N‐terminal and C‐terminal domains with an α/β fold. In DC301, hydrogen bonds are crucial for stabilizing interactions of the DC301 monomers 3. Thr191, Val192, Thr193, and Thr194 of one subunit of DC301 interact with Thr199, Thr194, Thr193, and Val192 of the other subunit via hydrogen bonds 3. The sequence alignment of DC301 to bmMTHFD (Fig. 1) indicated that Thr193, Val194, Thr195, and Ile196 of the *B. mori* residues interact with Thr201, Ile196, Thr195, and Val194 of the other subunit in a similar manner to DC301. These amino acid residues form β‐strand in the C‐terminal region and provide bridging hydrogen bonds in bmMTHFD that contribute to stabilizing the dimer of bmMTHFD. There are hydrogen bonds observed between the side chain of Thr193 in one subunit and side chain of Thr201 in the other subunit, main chain of Val194 of one subunit and main chain of Ile196 in the other subunit, side chain of Thr195 in one subunit and the counterpart in the other subunit, and main chain of Ile196 in one subunit and main chain of Val194 in the other subunit (Fig. 1).

**Table 1:** Data collection and refinement statistics. Values in parentheses indicate the highest‐resolution shell

Space group	*P*4_3_
Unit cell parameters (Å)	*a *= *b *=* *89.79, *c *=* *127.31
Resolution range (Å)	38.3–1.75 (1.78–1.75)
Total number of reflections	735 590
Number of unique reflections	100 729 (5028)
Multiplicity	7.3 (7.6)
*R*_merge_ a (%)	5.7 (48.7)
<*I>*/<σ(*I*)>	52.3 (6.15)
Completeness (%)	99.3 (100.0)
Refinement statistics	
Resolution range (Å)	38.3–1.75
Number of reflections	100 664
*R*_work_ b (%)/*R*_free_ c (%)	21.1/24.1
Root‐mean‐square deviations	
Bond lengths (Å)/bond angles (°)	0.007/0.869
Average *B*‐factors (Å^2^)/number of atoms	
Protein (Chain A, B)	32.4/2230, 33.5/2244
Small molecules^d^	37.5/32
Water	37.7/315
Ramachandran plot	
Favored region (%)	98.4
Allowed region (%)	1.23
Outliers (%)	0.35

a *R*_merge_ = ∑(*I* − <*I*>)/∑<*I*>, where *I* is the intensity measurement for a given reflection and <*I*> is the average intensity for multiple measurements of this reflection.

b *R*_work_ = ∑|*F*_obs_ − *F*_cal_|/∑*F*_obs_, where *F*_obs_ and *F*_cal_ are the observed and calculated structure‐factor amplitudes.

c The *R*_free_ value was calculated using only an unrefined, randomly chosen subset of reflection data (5%) that were excluded from refinement.

d Small molecules include sulfate ion and ethylene glycol.

### Cofactor‐binding site

The active site of DC301 contains a cofactor‐binding site and a substrate‐binding site 3. Superposition of structures between bmMTHFD and DC301 (PDB ID: 1A4I) revealed that the NADP^+^‐binding site of bmMTHFD could be composed of Arg166, Ser167, Arg199, and Ile242 (Fig. 4A). By using superposition of structures between bmMTHFD and hMTHFD2 (PDB ID: 5TC4) 24, we found that the amino acid residues Arg166, Ser167, Asn169, Arg199, Ile242, and Thr281 could be involved in NAD^+^ binding (Fig. 4B).

### Putative substrate‐binding sites

**Figure 3:**
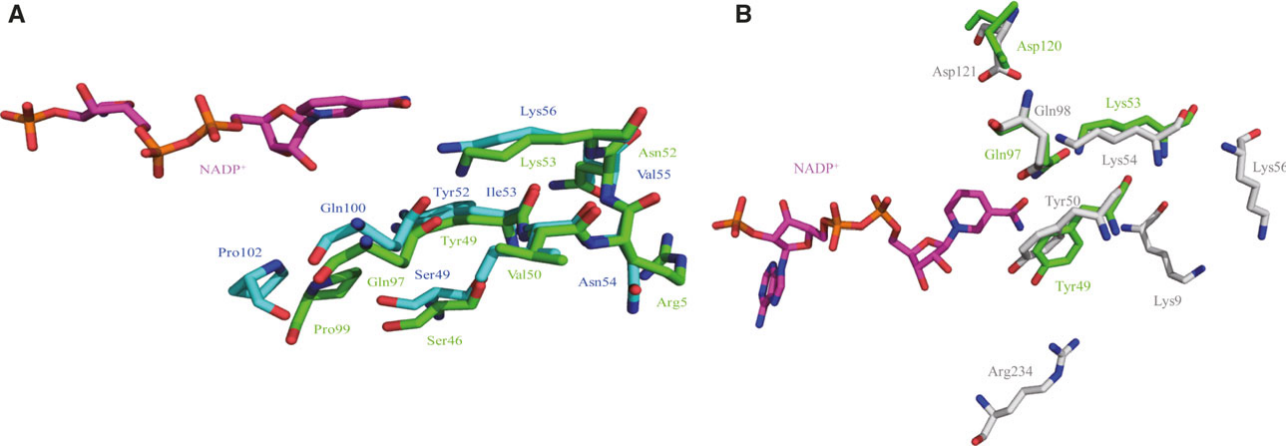
Superimposed structures of putative substrate‐binding site of bmMTHFD with DC301 (A) and *Escherichia coli* MTHFD (B). Structures of bmMTHFD, DC301, *E. coli* MTHFD, and NADP ^+^ are shown in green, cyan, gray, and magenta, respectively. Atoms for oxygen, nitrogen, and phosphorus are red, blue, and orange, respectively. Amino acid residues for bmMTHFD, DC301, and *E. coli* MTHFD are described in green, blue, and gray, respectively.

In DC301, Tyr52‐XXX‐Lys56 was identified as the YXXXK motif that plays a role in catalysis 3. The motif is also conserved in bmMTHFD as Tyr49‐XXX‐Lys53. Ser49, Gln100, and Pro102 may also play a role in catalysis 3. Superposition of DC301 with bmMTHFD revealed that the equivalent bmMTHFD residues were Ser46, Gln97, and Pro99 (Fig. 3A). Furthermore, the putative substrate‐binding site of *E. coli* MTHFD (PDB ID: 1B0A) is composed of Lys9, Tyr50, Lys54, Lys56, Gln58, Asp121, and Arg234 10. We identified four residues of bmMTHFD, Tyr49, Lys53, Gln97, and Asp120 that correspond to these in *E. coli* MTHFD 10 (Fig. 3B).

### Amino acid residues involved in substrate and cofactor binding

*Bombyx mori* MTHFD produced in bacteria was purified to homogeneity, yielding a single band in SDS/PAGE with a molecular size of ~ 32 000 Da. This was close to the estimated size based on the deduced amino acid sequence.

We identified Ser46, Tyr49, Lys53, Gln97, and Asp120 as amino acid residues crucial for catalysis. These amino acid residues are highly conserved within MTHFD as the substrate‐binding site (boxed in red, Fig. 1). To determine whether these residues were important for catalytic activity, we performed site‐directed mutagenesis. The resulting mutants were named S46A, Y49A, K53A, Q97A, and D120A and were purified from *E. coli* clones using the same method as for the wild‐type (WT) enzyme. A single band was obtained for each preparation of the mutant enzyme in SDS/PAGE. We examined optimum condition for bmMTHFD assay (Fig. S2). We determined the kinetic parameters of the mutant enzymes under the optimum condition (Fig. S2) and compared these with the kinetic parameters of the WT enzyme (Table 2). The catalytic efficiencies represented by *k*_cat_/*K*_m_ for MTHF with Y49A and D120A were insignificant (Table 2). The *k*_cat_/*K*_m_ for NADP^+^ was 8.6 × 10^−3^ mm ^−1·^s^−1^ for the WT bmMTHFD, which was 2.6‐, 1.4‐, 1.7‐, and 4.1‐fold higher than that for the S46A, K53A, Q97A, and D120A mutants, respectively (Table 2). The values of Y49A toward MTHF and NADP^+^ were not detected. In the case of NAD^+^, the *k*_cat_/*K*_m_ values for the mutants were similar to that of WT, although we were unable to detect the *k*_cat_/*K*_m_ of Y49A for 5,10‐methenyl‐THF, unlike the other mutants (Table 2).

**Table 2:** Comparison of kinetic data for D activity (A) and C activity (B) of bmMTHFD. The values of *K*_m_ and *V*_max_ are expressed as the mean with ± SD from three independent experiments. *K*_m_, *V*_max_, and *k*_cat_/*K*_m_ are expressed as mm, mm·min^−1^, and mm ^−1·^s^−1^, respectively. Kinetic parameters were measured with a nonlinear least squares data fit to the Michaelis–Menten equation under the assay conditions described in Materials and methods. Kinetic data of MTHF were measured in the presence of NADP^+^. ND means not detected

(A) D activity						
	WT	S46A	Y49A	K53A	Q97A	D120A
MTHF						
*K*_m_	0.51 (± 3.3 × 10^−2^)	0.19 (± 4.8 × 10^−3^)	ND	1.1 (± 1.8 × 10^−2^)	0.81 (± 2.8 × 10^−2^)	ND
*V*_max_	1.6 × 10^−3^ (± 3.7 × 10^−5^)	6.9 × 10^−4^ (± 6.2 × 10^−6^)	ND	4.1 × 10^−3^ (± 6.9 × 10^−5^)	5.8 × 10^−3^ (± 1.8 × 10^−4^)	ND
*k*_cat_/*K*_m_	0.043	0.049	ND	0.051	0.097	ND
NADP^+^						
*K*_m_	0.22 (± 1.0 × 10^−2^)	0.98 (± 1.3 × 10^−1^)	ND	0.62 (± 6.5 × 10^−2^)	0.33 (± 5.8 × 10^−2^)	2.0 (± 1.4 × 10^−1^)
*V*_max_	6.9 × 10^−4^ (± 1.4 × 10^−5^)	1.1 × 10^−3^ (± 2.0 × 10^−5^)	ND	9.8 × 10^−4^ (± 1.4 × 10^−4^)	6.9 × 10^−4^ (± 3.8 × 10^−5^)	1.0 × 10^−3^ (± 2.5 × 10^−5^)
*k*_cat_/*K*_m_	8.6 × 10^−3^	3.3 × 10^−3^	ND	6.2 × 10^−3^	5.2 × 10^−3^	2.1 × 10^−3^
NAD^+^						
*K*_m_	0.27 (± 2.0 × 10^−2^)	0.45 (± 8.5 × 10^−2^)	ND	0.30 (± 3.0 × 10^−2^)	0.47 (± 8.6 × 10^−2^)	2.0 (± 9.0 × 10^−2^)
*V*_max_	6.2 × 10^−4^ (± 1.7 × 10^−5^)	8.4 × 10^−4^ (± 3.0 × 10^−5^)	ND	7.3 × 10^−4^ (± 3.0 × 10^−6^)	9.7 × 10^−4^ (± 4.1 × 10^−5^)	1.0 × 10^−3^ (± 5.9 × 10^−5^)
*k*_cat_/*K*_m_	7.0 × 10^−3^	5.6 × 10^−3^	ND	6.6 × 10^−3^	5.1 × 10^−3^	1.9 × 10^−3^

(B) C activity						
	WT	S46A	Y49A	K53A	Q97A	D120A
*K*_m_	0.13 (± 6.0 × 10^−3^)	0.16 (± 1.0 × 10^−2^)	ND	0.16 (± 1.5 × 10^−2^)	0.019 (± 2.5 × 10^−3^)	0.23 (± 2.5 × 10^−2^)
*V*_max_	6.4 × 10^−4^ (± 2.5 × 10^−5^)	6.3 × 10^−4^ (± 1.5 × 10^−5^)	ND	5.7 × 10^−4^ (± 2.5 × 10^−5^)	1.5 × 10^−4^ (± 5.0 × 10^−6^)	8.2 × 10^−4^ (± 4.5 × 10^−5^)
*k*_cat_/*K*_m_	0.13	0.11	ND	0.097	0.22	0.097

## Discussion

This study aimed to detect the presence of one‐carbon metabolism in the silkworm *B. mori* and to analyze whether MTHFD is relevant for insecticide design. The amino acid sequence of bmMTHFD revealed high homologies to those of mitochondrial MTHFD2 (49%) and MTHFD2L (51%). However, bmMTHFD did not possess a predicted N‐terminal mitochondrial targeting sequence (Fig. 1).

According to substrate specificity, MTHFD proteins are divided into mono‐, bi‐, and trifunctional proteins. For instance, MTHFD1 is a trifunctional enzyme 25, MTHFD2 is a bifunctional enzyme 26, and MTHFD1L is a monofunctional enzyme 9. Our experiments revealed that bmMTHFD is bifunctional, exhibiting D and C activities (Table 2). In DC301, Tyr52‐XXX‐Lys56 and/or Ser49, Gln100, and Pro102 were proposed as C active site. Since we found the corresponding residues in bmMTHFD to be Tyr49‐XXX‐Lys53 and/or Ser46, Gln97, and Pro99 3, we predicted that bmMTHFD possesses the C domain (Fig. 1). The amino acid residues required for S activity are located in the S domain of the 70 kDa MTHFD1. However, bmMTHFD lacks the S domain for S activity (Fig. 1).

Elucidation of the bmMTHFD tertiary structure identified a globular shape that was similar to those of other known MTHFDs. In addition, the conserved amino acid residues are critical for stabilizing the interaction of bmMTHFD monomers. Our study indicates that Thr193, Val194, Thr195, Ile196, and Thr201 are involved in hydrogen bond interactions, suggesting that these amino acid residues are involved in maintaining the homodimer of bmMTHFD.

Our findings show that bmMTHFD contains the catalytic site where the substrate binds in the N‐terminal domain and displays cofactor binding in the C‐terminal domain, like other MTHFDs. bmMTHFD is composed of 10 α‐helices and nine β‐strands, and their location is highly conserved in DC301 (Fig. 1).

**Figure 4:**
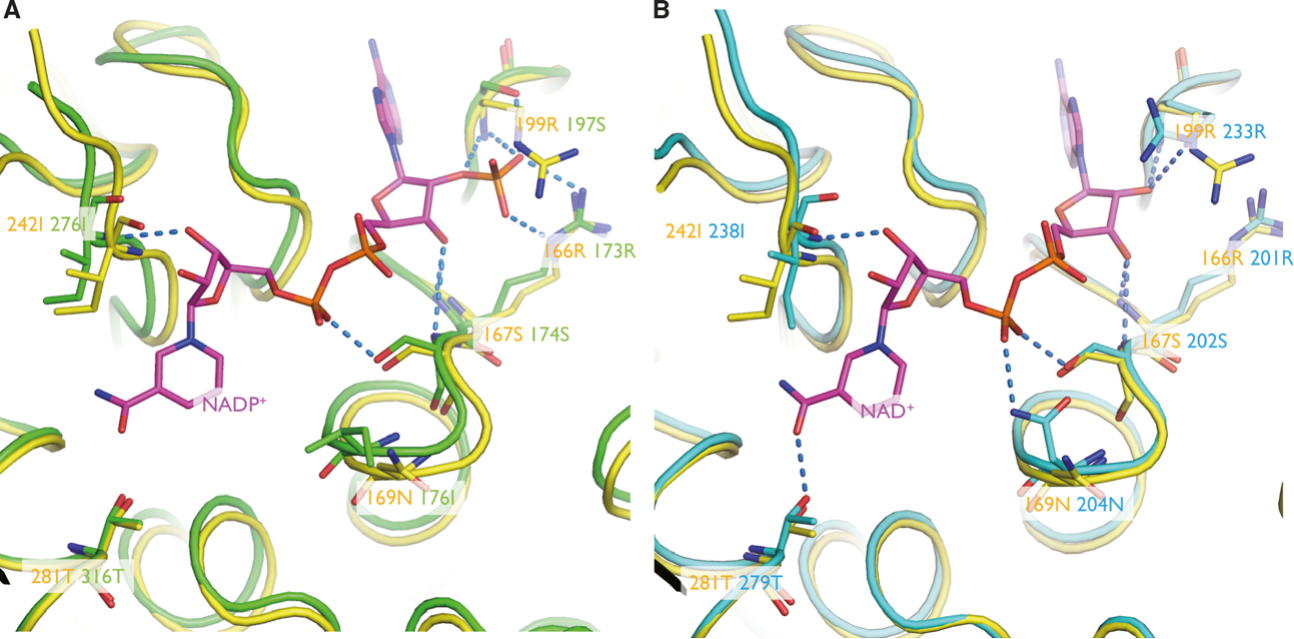
Cofactor‐binding site. (A) The structures of bmMTHFD, DC301, and NADP ^+^ are shown in yellow, green, and magenta, respectively. Residue labels for bmMTHFD and DC301 are in yellow and green, respectively. (B) The structures of bmMTHFD, hMTHFD2, and NAD ^+^ are shown in yellow, cyan, and magenta, respectively. Residue labels for bmMTHFD and hMTHFD2 are in yellow and cyan, respectively. The sulfate ion is indicated in yellow and labeled in black. Color scheme: oxygen, red; nitrogen, blue; sulfur, yellow. The blue dots show hydrogen bonds.

The *K*_m_ value of bmMTHFD was also found to be similar to those for *Pseudomonas aeruginosa* (26 μm for MTHF and 176 μm for NADP^+^) 27, and *Trypanosoma brucei* (35 μm for MTHF and 70 μm for NADP^+^) 28. bmMTHFD uses either NADP^+^ or NAD^+^ for catalysis with similar *k*_cat_/*K*_m_ values (Table 2). Similar results were obtained with mammalian MTHFDs, where hMTHFD2 and mMTHFD2L were reported to utilize both NADP^+^ and NAD^+^ 7, 8. Superimposition between bmMTHFD and DC301 requiring NADP^+^ indicated that a single cofactor bound to the site contained Arg166, Ser167, Arg199, and Ile242 (Fig. 4A). Superimposition between bmMTHFD and hMTHFD2 requiring NAD^+^ showed that Arg166, Ser167, Asn169, Arg199, Ile242, and Thr281 were present in bmMTHFD in a dinucleotide‐binding motif (Fig. 4B). In bmMTHFD, the motif (165GRSRQVG171) does not conform to the classical GXGXXG fingerprint pattern. The sequence comparisons did not suggest the amino acid residues that may be involved in NAD^+^ utilization. Cocrystallization with NADP^+^ or NAD^+^ would allow improved prediction of specificity.

Tyr52, Lys56, Gln100, and Asp125 in hMTHFD1 have been identified as critical for its activity 29. Superimposition between DC301 and bmMTHFD revealed that the corresponding residues in bmMTHFD were Tyr49, Lys53, Gln97, and Asp120, respectively. Tyr52 (bmMTHFD Tyr49) was implicated in substrate binding, and Lys56 (bmMTHFD Lys53) and Gln100 (bmMTHFD Gln97) were proposed to work together in establishing an environment for hydride transfer 3, 29, 30. Asp125 (bmMTHFD Asp120) serves in substrate binding. Mutations at Tyr49 eliminated affinities to MTHF and 5,10‐methenyl‐THF. Mutagenesis of Asp120 influences the D activity. The results are consistent with a key role for Tyr52, and Asp125 in hMTHFD1 to correctly bind the folate substrate. Since MTHFDs have an aspartate residue at the 125 position, the carboxylate is invariant. The side chain would then be in position to interact with the edge of the pterin ring at N3 and at the exocyclic amine of C2 of pterin 31. In bmMTHFD, the electron density from Pro44 to Ser46 of A chain was disordered. In DC301, there is a hydrogen‐bonding network near these amino acid residues 3. To examine whether Ser46 contributes to bmMTHFD activity, we mutated the amino acid residue to Ala. Subsequent mutagenesis results showed that their kinetic parameters were not changed significantly (Table 2). In *E. coli* MTHFD, mutation of Asp121 (bmMTHFD Asp120) decreases the *k*_cat_/*K*_m_ values toward MTHF to 0.17%, while the kinetic parameters of mutating Tyr50 (bmMTHFD Tyr49) could not be determined, due to very low activities 32. The results indicate that the catalytic mechanism could be similar to that in *E. coli* MTHFD.

In conclusion, this study identified and biochemically characterized bmMTHFD, a MTHFD in the silkworm *B. mori*. To the best of our knowledge, this study is the first to report an MTHFD in insects. Furthermore, we identified bmMTHFD amino acid residues that are likely to play roles in catalysis. We are currently attempting the cocrystallization of bmMTHFD with a suitable substrate analogue conjugate to aid in the determination of amino acid residues involved in bmMTHFD catalysis. Our findings provide insight into the mechanism underlying bmMTHFD activity and potentially that of other insects and may therefore facilitate the development of more effective and safe insecticides.

## Conflict of interest

The authors declare no conflict of interest.

## Author contributions

MRH, AH, and KY designed experiments. MRH, AH, AH, and KY performed experiments. MRH, AH, AN, AH, SF, and WC analyzed the data. AN, SF, and KY wrote the manuscript.

## Supporting information


**Fig. S1.** Superdex 200 analytical gel filtration. Protein standards (closed circle) include thyroglobulin (670 kDa), globulin (158 kDa), ovalbumin (44 kDa), and myoglobin (17 kDa). The plot of log molecular weight vs elution volume Wt with an *R* ^2^ value of 0.999. Closed triangle indicates position of bmMTHFD.

**Fig. S2.** Enzymatic properties of bmMTHFD. The enzymatic properties of bmMTHFD were analyzed using 5, 10‐methylenetetrahydrofolate as substrate and NADP^+^ as cofactor. The MTHFD activity was assayed under standard conditions, as described in Experimental Procedures, unless otherwise indicated. The maximum value obtained was set to 100%. Data represent the mean with ± SD from three independent experiments. (A) pH stability was assessed by preincubation of the enzyme solution at various pH values at 4 °C for 24 h before the residual activity was assayed. (B) Thermostability was determined by preincubation of the enzyme solution at various temperatures at pH 6 for 30 min before the residual activity was assayed. (C) Optimum pH levels for the activities were assayed at 28 °C using citrate‐phosphate‐borate buffer at various pH value.
