# Supplementary figures and images for: Molecular structure of a 5,10‐methylenetetrahydrofolate dehydrogenase from the silkworm Bombyx mori
Source: FEBS Open Bio. 2019 Feb 26;9(4):618–28. doi: 10.1002/2211-5463.12595 (PMC6443876; doi:10.1002/2211-5463.12595)

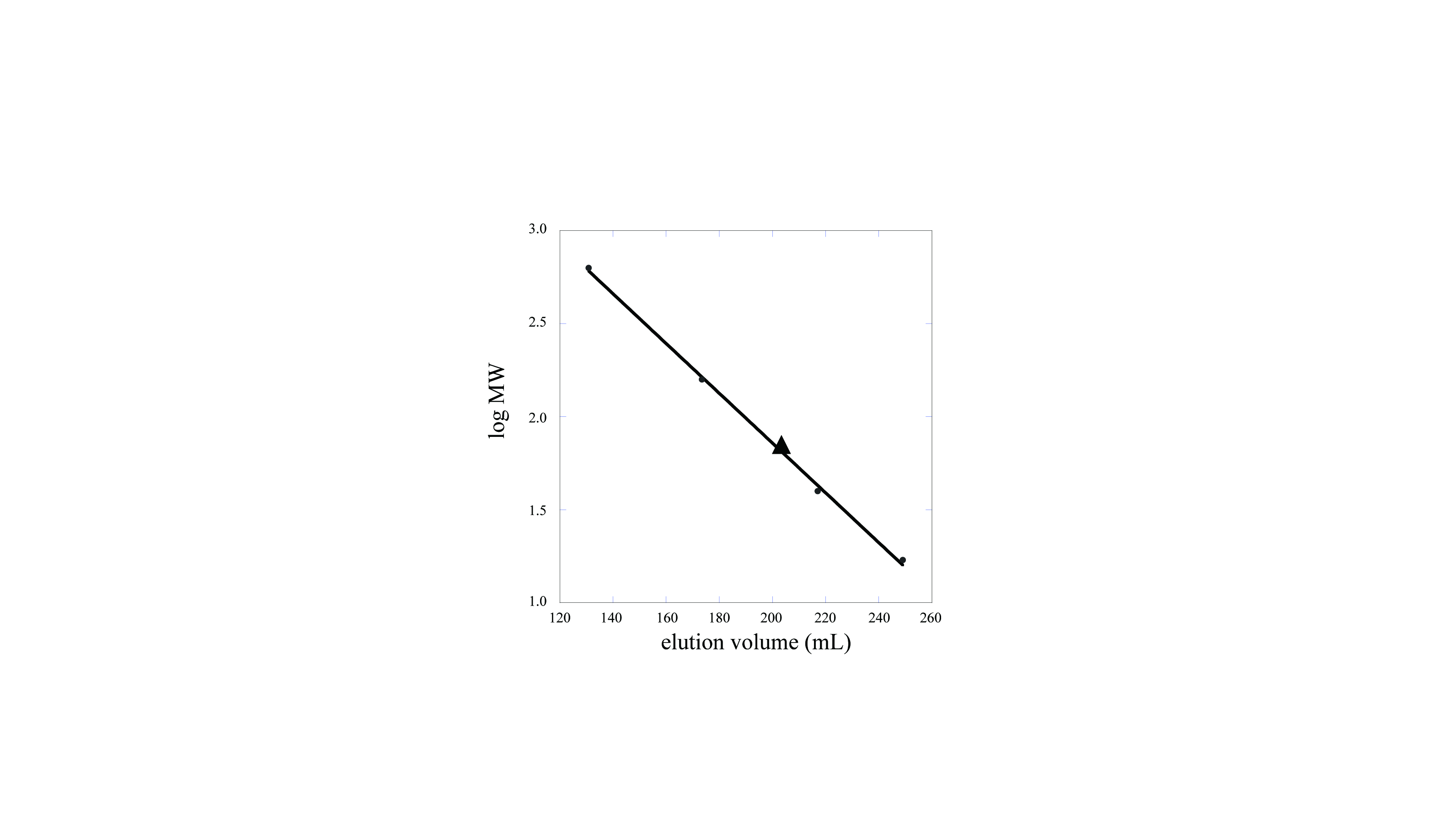

Supplement: Supplementary file 1 — Fig. S1. Superdex 200 analytical gel filtration. Protein standards (closed circle) include thyroglobulin (670 kDa), globulin (158 kDa), ovalbumin (44 kDa), and myoglobin (17 kDa). The plot of log molecular weight vs elution volume Wt with an R 2 value of 0.999. Closed triangle indicates position of bmMTHFD. [file FEB4-9-618-s001.tif]

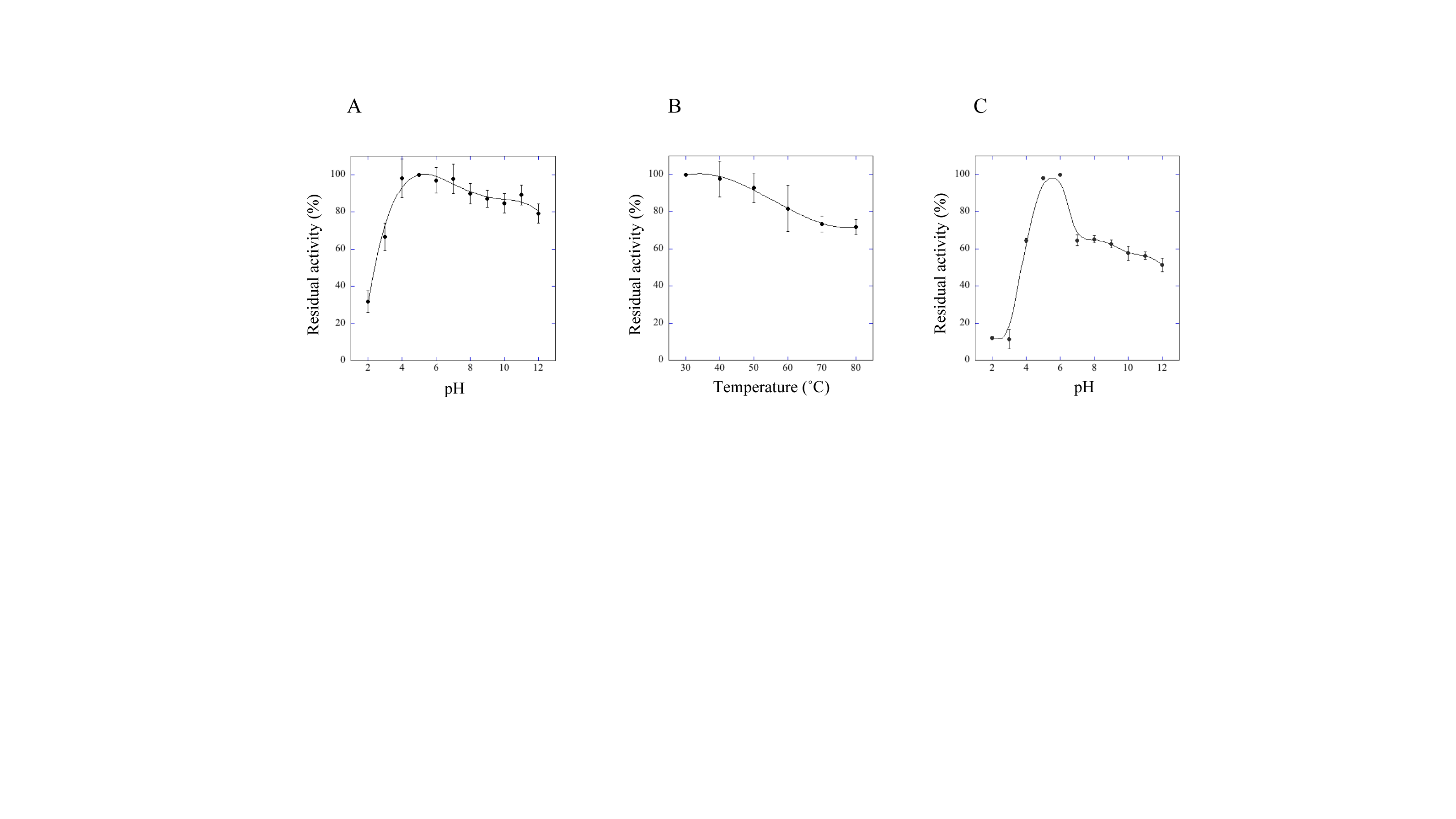

Supplement: Supplementary file 2 — Fig. S2. Enzymatic properties of bmMTHFD. The enzymatic properties of bmMTHFD were analyzed using 5, 10‐methylenetetrahydrofolate as substrate and NADP+ as cofactor. The MTHFD activity was assayed under standard conditions, as described in Experimental Procedures, unless otherwise indicated. The maximum value obtained was set to 100%. Data represent the mean with ± SD from three independent experiments. (A) pH stability was assessed by preincubation of the enzyme solution at various pH values at 4 °C for 24 h before the residual activity was assayed. (B) Thermostability was determined by preincubation of the enzyme solution at various temperatures at pH 6 for 30 min before the residual activity was assayed. (C) Optimum pH levels for the activities were assayed at 28 °C using citrate‐phosphate‐borate buffer at various pH value. [file FEB4-9-618-s002.tif]
